# The Uneven Distribution of Medically Underserved Areas in Chicago

**DOI:** 10.1089/heq.2020.0023

**Published:** 2020-12-30

**Authors:** Sage J. Kim, Caryn E. Peterson, Richard Warnecke, Richard Barrett, Anne Elizabeth Glassgow

**Affiliations:** ^1^ Divisions of Health Policy and Administration, School of Public Health, University of Illinois at Chicago, Chicago, Illinois, USA.; ^2^ Divisions of Epidemiology and Biostatistics, School of Public Health, University of Illinois at Chicago, Chicago, Illinois, USA.; ^3^ Institute for Health Research and Policy, University of Illinois at Chicago, Chicago, Illinois, USA.; ^4^ Department of Sociology, University of Illinois at Chicago, Chicago, Illinois, USA.; ^5^ Department of Medicine, University of Illinois at Chicago, Chicago, Illinois, USA.

**Keywords:** medically underserved areas, racial disparity, safety net services, Federally Qualified Health Centers

## Abstract

**Purpose:** Safety net health services, such as federally funded health clinics, are interventions that aim to mitigate inequality in resource distribution, thus primarily clustered in poor areas with lack of access to health care. However, not all neighborhoods with the most needs benefit from safety net health services. In this article, we explore the distribution of a federally funded health service intervention designed to serve impoverished areas, the medically underserved areas (MUAs), and the relationship between MUA designation and neighborhood sociodemographic characteristics.

**Methods:** We explore the spatial distribution of MUAs. The 2010 U.S. census data including 868 census tracts in Chicago were used for the analysis. We then examined the likelihood of being designated as an MUA using census tract level neighborhood demographic variables.

**Results:** We found that the likelihood of obtaining MUA designation increases for neighborhoods with higher levels of poverty, the likelihood of being designated as an MUA begins to decline beyond the tipping point, whereas the proportion of black residents continues to increase. In census tracts that were eligible but not designated, there was a greater proportion of black residents compared with white residents (*p*<0.01). The census tracks also had higher mean disadvantage scores (*p*<0.01) and lower social capital (*p*<0.01). Furthermore, MUA eligible areas that were not designated as MUAs were predominantly black neighborhoods in poverty.

**Conclusion:** Studies have documented that receiving MUA designation substantially reduces disparities in access to health care, and yet, our study finding indicates that the most racially segregated poor neighborhoods are excluded from the benefits of having such federal health safety net program. Seemingly race-neutral safety net health services may still be distributed in a way that perpetuates racial inequality in health.

## Introduction

### Safety net programs and social capital

Safety net programs are, by design, located in underserved areas to provide necessary services for those who lack access to resources.^1^ Although overall safety net programs have declined in the neoliberal era,^2,3^ welfare services continue to mitigate the uneven spatial distribution of resources. For example, Small and Stark document that the likelihood of having public childcare centers, a safety net program, was higher in poor neighborhoods, compared with affluent neighborhoods.^1^

Safety net programs are typically delivered through local service providers.^4,5^ Unlike individual cash assistance programs such as food stamps, social service programs are place based.^4^ Thus safety net programs represent neighborhood institutions that shape how people gain access to health care and social services in poor communities^5,6^ and mitigate the effect of neighborhood poverty on individual health outcomes.^7–9^

Although safety net programs are designed to provide services to underserved areas, not all disadvantaged neighborhoods equally benefit from such programs. The distribution of safety net programs is often influenced by contextual factors, particularly neighborhood social capital.^10^ Social capital refers to neighborhood capacity to deal collectively with shared issues.^6,11^ Strong local organizations enhance social capital by mediating bonding among individuals, bridging individuals and organizations, and linking to external resources.^12^ As a result, neighborhoods with weak social capital tend to have difficulty drawing external resources.

### Medically underserved areas

Designation as a medically underserved area (MUA) is one example of organizational mediation in health care systems insofar as they mitigate the impact of neighborhood disadvantage on health. An MUA is a geographic area designated as lacking access to primary care services^13^ and is eligible for enhanced reimbursement from Medicare and Medicaid for primary care services through Federally Qualified Health Centers (FQHCs). The geographical area can be a whole county, a group of neighboring counties, a group of urban census tracts, or a group of county or civil divisions.^13^ The Health Resources and Services Administration (HRSA) is responsible for the evaluation of eligibility and designation of MUA based on the Index of Medical Underservice (IMU) score of a service area.^13^ The IMU includes four variables, each with a standardized contribution of 25% to the overall score: (1) the ratio of primary care physicians per 1000 residents, (2) the infant mortality rate, (3) % of the population with incomes below the poverty level, and (4) % of the population age 65 years or older. The IMU score can range from 0 to 100, where lower IMU scores indicate areas of underservice. Areas with an IMU of 62 or lower are eligible for MUAs. Once designated, MUAs are eligible for the establishment of FQHCs.

Although FQHCs represent only one component of the health care safety net, they are pivotal players in providing essential primary care.^14^ In 2015, FQHCs served close to 24.3 million individuals through almost 97 million patient encounters.^15^ Of the patients receiving care in FQHCs, 77% were living below the poverty level and 58% were on Medicaid.^16^ Nationally, $3.7 billion federal dollars were allocated for FQHCs in 2014.^17^ Furthermore, several studies have examined the impact of MUAs and FQHCs on access to health care on having a usual source of care and more physician visits.^18,19^ Thus, government interventions such as MUAs have a potential impact on eliminating health disparities.

To date, most neighborhood research on access to care has focused on comparisons between affluent and impoverished areas. However, it may not simply be the neighborhood economic characteristics that determine access, as much as the availability of government services or lack thereof. For example, poor residents who are relocated into wealthier areas have difficulty accessing social services^1^ because these government services targeted to help the poor tend to cluster in underserved neighborhoods and not in wealthier areas. However, whether all poor neighborhoods are equally likely to receive health services programs is not well understood. Although all MUAs are, by definition, poor and underserved,^13^ not all poor areas are designated as MUAs. Although safety net programs are intended to serve impoverished areas, these neighborhood interventions may not be distributed solely based on the level of need.^4^ The first step to being designated as an MUA is to meet the eligibility criteria; however, a community organization or facility that wishes to receive MUA status are also required to submit a successful application to the State Primary Care Office. Thus, understanding the characteristics determining eligible neighborhoods to apply for government health resources is an important next step toward health equity.

### Conceptual model and study objectives

**FIG. 1.**
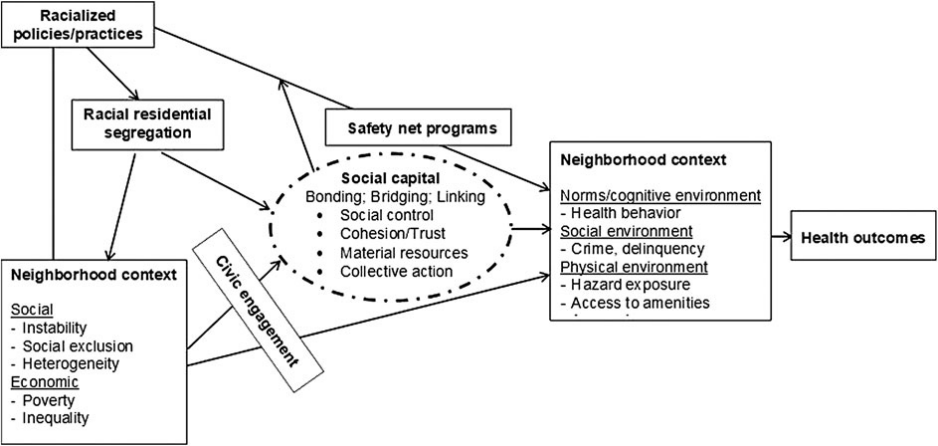
Conceptual model for the effects of neighborhood context and social capital on health.

Figure 1 provides the relationships between neighborhood context (including social and economic disadvantage), civic engagement, social capital, and health outcomes. We argue that neighborhoods with a higher level of social capital are more likely to obtain MUA designation. Social and economic conditions and the level of civic engagement influence social capital. In addition, we assume that racial residential segregation has detrimental effects on not only neighborhood social and economic conditions, but also social capital. Furthermore, social capital can influence how policies are implemented. For the purpose of our analysis, we consider whether a community can obtain safety net programs, such as MUA and FQHCs. Combined, all these neighborhood factors determine the type of neighborhood context, including norms supporting healthy behavior, access to health care and social services, and crime and delinquency. Ultimately, neighborhood context affects health outcomes.

In this article, we first explore the spatial distribution of MUA designation and FQHCs. Second, we explore the relationship between neighborhood contextual factors and the likelihood of obtaining MUA designation. Finally, we examine the effect of area-level racial/ethnic composition on the likelihood of the MUA designation.

## Methods

### Data and measures

We used the spatial distribution of MUA designation and FQHCs in Chicago neighborhoods eligible for MUA designation (IMU score of ≤62) to evaluate differences between MUA-eligible neighborhoods that received MUA status and those that did not. First, we identified areas that are underserved and subsequently designated as MUA based on the IMU criteria. Second, to identify areas that are underserved, but not designated as MUA, we received IMU scores for all census tracts in Chicago from the MUA administrator for the state of Illinois. These scores determined an area's current MUA eligibility (email communication with Nolan Nosari, February 28, 2009). We then updated the MUA status using the HRSA “MUA Find” website as of 2018.^20^ We also updated the locations of current FQHCs in Chicago using the HRSA “Find a Health Center.”^21^

Neighborhoods with the IMU score >62 are not eligible to apply for MUA. These areas are relatively affluent and have lower levels of health risk and access to care burden. Areas with an IMU score 62 or lower are considered to be MUA eligible. All census tracts in Chicago (*N*=868) were categorized into three categories of MUA status: (1) affluent neighborhoods that are not eligible for MUA designation (“ineligible”), (2) underserved areas that have been designated as MUAs (“eligible/designated”), and (3) underserved neighborhoods that are eligible but not designated as MUAs (“eligible/nondesignated”).

Socioeconomic and demographic factors at the census tract level were derived from the U.S. Census, American Community Survey (ACS) 5-year estimates between 2013 and 2017. Variables included were: % residents living below 100% federal poverty line, % blacks, % Hispanics, median household income, % unemployed, % residents with less than high school education, and % residents older than 65 years of age. Because % unemployed is calculated based on only those in the labor market, this measure does not take into account those who are out of the labor market entirely. To minimize such bias, we calculated the employment ratio, which is the proportion of people who are working among all working-age individuals between 16 and 65 years of age.^22^

To quantify the level of disadvantage, we used the index of concentrated disadvantage that are widely used to measure the effects of neighborhood context on disparities.^23–26^ The index accounts for: % poverty, % blacks, % less than high school education, % female-headed households, and median household income. We also geocoded addresses of FQHCs and calculated the average number of FQHCs per census tract. To examine the coverage of FQHCs for the poor, we estimated the average number of people living below the poverty line per health center within each census tract.

Social capital was measured using three components: social interaction, civic engagement, and economic potential. Social interaction included interaction, stability, and diversity, and economic potential included commercial vitality, buying power, neighborhood investment, and workforce (Table 1). This study was reviewed by the University of Illinois at Chicago Institutional Review Board (IRB) and determined to be non-human subject (IRB# 2020-1584).

**Table 1.** Social Capital Index Components and Variables

Components and sub-indices	Variables
Social interaction^a^	
Interaction	% Households speak language other than English (inverse)
	% Single person households (inverse)
	% Households with one or more adults not in the labor force
Stability	% Households resided in same home 5 years
	% Foreign born residents entered into tract within 5 years
Diversity	% Residents of largest race/ethnic group (Black, Hispanics, White, Other)
	% Residents of largest age group (0–24, 25–44, >45 years)
	% Households in largest income group (<$35,000, $35,000–$75,000, >$75,000)
Civic engagement	
Civic engagement	% Eligible residents voted in general elections 2014, 2016, 2018^b^
	Number of nonprofit organizations per 1000 residents^c^
Economic potential	
Commercial vitality	Number of businesses per 1000 residents^d^
	Total amount of small business loans (<$1 million) per 1000 residents^e^
Buying power^a^	Median household income
	% Households spent >30% of income on housing
Neighborhood investment	Number of mortgages originated per dwelling unit^f^
	Number of home improvement loans per occupied dwelling unit^f^
	% Occupied dwelling units^a^
Workforce^a^	% Residents 25 years and older with more than high school education
	% Employed
	Employment-population ratio among working age individuals 16–64 years of age

Data source

^a^ American Community Survey (ACS) 5 years estimates for 2013–2017, U.S. Census Bureau.

^b^ Chicago Board of Election Commissioners.

^c^ National Center for Charitable Statistics.

^d^ Chicago Data Portal.

^e^ Community Reinvestment Act Data, 2013–2017.

^f^ Home Mortgage Disclosure Act Data, 2013–2017.

### Statistical analysis

The distribution of MUAs and the location of FQHCs were visualized using ArcGIS 10.5, Geographic Information Systems (GIS) software. Descriptive statistics were used to examine the relationship between the MUA status and % blacks as well as % poverty at the census tract level. Differences in the distribution of neighborhood characteristics by MUA status were assessed using chi-square test statistics. Logistic regression was conducted to estimate the likelihood of a neighborhood being MUA-eligible and designated. Stata^®^ 15 was used to conduct descriptive and regression analyses.

## Results

### Distribution of MUAs

**FIG. 2.**
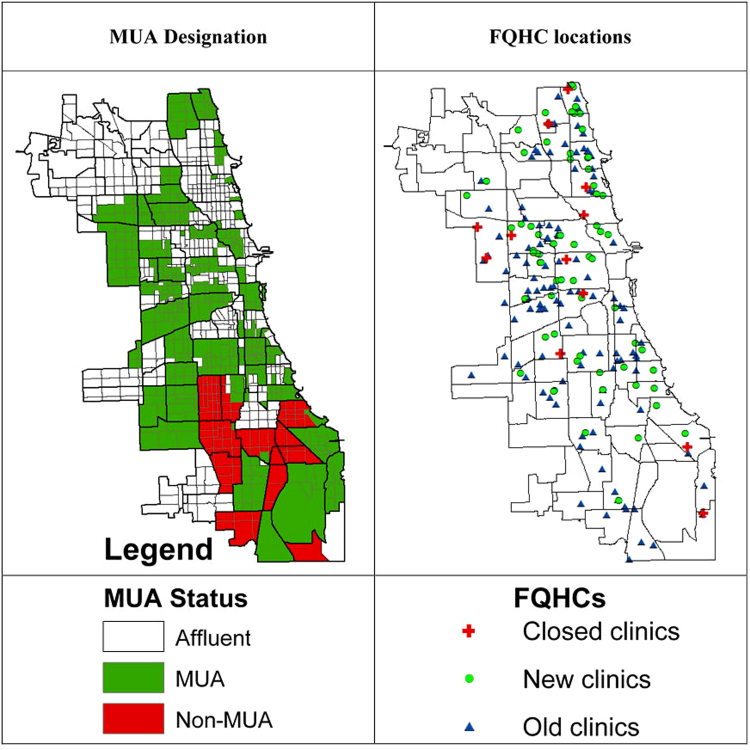
Distribution of MUA and FQHCs in Chicago. *Gray lines* indicate census tracts and *dark lines* indicate Chicago community areas. *Unshaded areas* indicate affluent areas that are not eligible for MUA; *Green areas* indicate poor areas that are eligible and designated as MUA; and *Red areas* indicate poor areas that are eligible but not designated as MUA. FQHCs, Federally Qualified Health Centers.

Figure 2 presents the distribution of MUAs and FQHCs in Chicago. The first map shows the distribution of MUAs in Chicago. There were 391 census tracts in affluent neighborhoods, thus designation for MUA. These areas were mostly located on the north and northwest side and in downtown areas. The 390 census tracts that were eligible and designated for MUA status were clustered on the far north and west side of Chicago. The 87 census tracts that were eligible for MUA status (IMU scores: 48.6–61.7) but not designated as an MUA were predominantly on the south side of Chicago. Census tracts that are eligible for MUA status but not designated as MUAs were predominantly black and poor.

The second map shows the locations of previous clinics that existed in 2009, and newly opened and closed clinics since 2009. There are a total of 187 clinics in Chicago. Of those, 131 clinics existed before 2009. Since 2009, 12 clinics were closed and 68 new clinics were opened.

Table 2 provides neighborhood characteristics by MUA status. Census tracts that were MUA eligible but not designated, compared with MUA designated tracts, had a greater proportion of black residents, lower proportion of Hispanic residents, higher mean disadvantage scores, fewer FQHCs and newly added clinics, and lower economic potential, and social capital index, at *p*<0.01 for all comparisons.

**Table 2.** Neighborhood Characteristics by MUA Status, Chicago (*N*=456 Tracts)

	Mean comparison by MUA status			
	Chicago	MUA	Non-MUA	p
Sociodemographics				
Number of census tracts	799	365	91	—
Total population	2,683,422	1,252,783	259,226	—
% Black	36.4	40.3	92.5	<0.01
% Hispanic	25.9	34.5	4.1	<0.01
% White	30.6	19.4	2.0	<0.01
% poverty	22.3	26.8	30.0	<0.05
Disadvantage score	0.0	0.30	0.92	<0.01
Uninsured 19–64 years	18.1	21.8	19.7	n.s.
Total number of FQHCs	187	112	10	—
Mean number per tract	0.2	0.3	0.1	<0.01
Newly added clinics	68	41	3	<0.05
FQHCs per 100,000 poor	39.6	40.8	15.5	<0.05
Social capital index	58.4	54.8	50.3	<0.01
Social interaction	65.4	65.7	64.7	<0.05
Economic potential	60.0	55.0	46.5	<0.01
Civic engagement	54.5	51.8	49.3	n.s.

FQHCs, Federally Qualified Health Centers; MUA, medically underserved area; Non-MUA, MUA-eligible but not designated; n.s., not significant.

Table 3 presents logistic regression models estimating the likelihood of being MUA designated among census tracts that were eligible for MUA. Variables concerning social, economic, civic engagement, and racial composition were entered into the model stepwise. Across all models, MUA designated census tracts were more likely to be diverse and stable. Neighborhoods with a greater number of community organizations per 1000 residents were more likely to be MUA designated. After controlling for factors relating to social interaction, economic potential, and civic engagement, neighborhoods with a lower proportion of black residents were more likely to receive MUA designation.

**Table 3.** Multivariable Models Estimating the Likelihood of Being MUA Designated Among All Eligible Tracts (*N*=456 Tracts)

MUA=1 Versus Non-MUA=0				
	Model I	Model II	Model III	Model IV
	Exp (B)			
Explanatory variable	N=455			
Social interaction				
Community diversity	1.66^**^	1.77^**^	1.85^**^	1.51^**^
Interaction potential	0.82^*^	0.98	0.96	1.07
Stability	0.63^**^	0.69^*^	0.69^*^	0.71^*^
Economic potential				
Buying power		0.96	0.95	0.88^*^
Neighborhood investment		0.91^*^	0.90^*^	0.93
Work force participation		1.10	1.08	1.08
Commercial vitality		1.10^**^	1.09^**^	1.03
Civic engagement				
Voting turnout			0.97	1.00
Number of NGOs per 1000			1.44^**^	1.71^**^
Racial segregation				
Black neighborhoods				-
Other neighborhoods				33.95^**^
−2 log likelihood	374.99	357.32	348.83	297.99
Pseudo R2	0.177	0.215	0.234	0.346

^*^ *p*<0.05.

^**^ *p*<0.01.

NGOs, Non-Governmental Organizations.

**FIG. 3.**
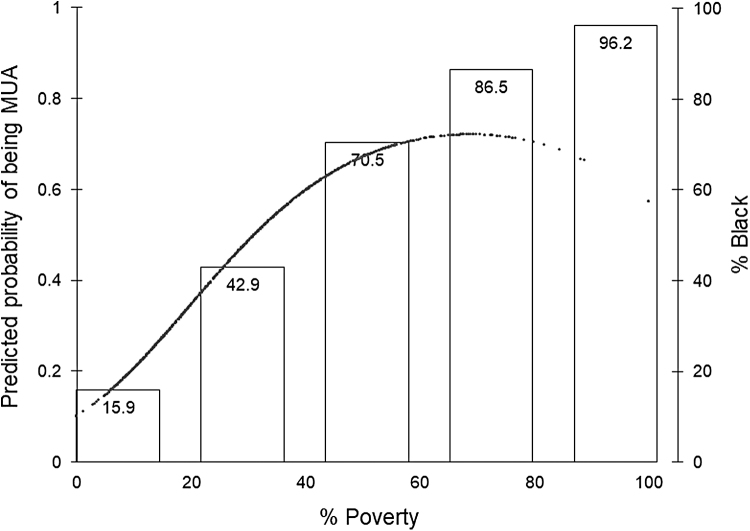
Predicted probability of being MUA and the proportion of *black* residents, by percent of residents living below the poverty line. The *line* represents the probability of being MUA designated; The *bar graph* represents the percentage of Black residents.

Figure 3 provides the predicted probability of being designated as an MUA in relation to % poverty and % black within the poverty quintile. The relationship between poverty and the probability of being MUA is nonlinear. As expected, census tracts with a lower poverty rate had a lower probability of being designated as MUA. As % poverty increased, the probability of being in an MUA also increased. However, at the poverty level of 68.9% the likelihood of obtaining MUA designation began to decline. On the contrary, the mean % black steadily increased by % poverty quintile ordinal categories. Neighborhoods with the highest percentage of poverty also had the highest percentage of black residents. Moreover, those areas were more likely not to have MUA designation.

## Discussion

We found that MUAs and FQHCs were more likely to be located in underserved neighborhoods in Chicago. Scholars have documented the importance of neighborhood service programs in poor areas and residents' reliance on them. For example, participants in the Moving to Opportunity (MTO) experiment who moved out of their original poor neighborhoods with housing vouchers^27^ were shown to have difficulty accessing services because their new better-off neighborhoods lacked safety net services.^28^ Similarly, Warnecke et al. found that neighborhood contextual factors including MUA designation were significantly associated with the late-stage diagnosis of breast cancer.^29^ These findings support the role of safety net programs in improving health. However, MUA designation in Chicago illustrates that not all underserved areas have equal access to federally funded services that mediate resource flow. To a certain extent, the probability of being MUA designated increased as the poverty level increases. However, the level of poverty beyond the tipping point was associated with a lower likelihood of having MUA. Furthermore, these neighborhoods with the highest poverty rates were exclusively black communities. This finding confirms previous scholars' argument that the combined effects of poverty and segregation are more than the separate effects of poverty and racial segregation.^30–34^ Wilson, for example, suggests that the “concentration effects” of social and economic changes in the extreme poverty areas (p. 46) result in a myriad of social, economic, and behavioral dislocations, which further result in the creation of “the underclass.”^35^

One of the enduring debates concerning inequality is whether race-specific or nonracial class-based policies are more appropriate for addressing the issues of race and urban poverty.^36^ However, as Squires and Kubrin conclude, even nonrace-specific policy decisions can have racial implications. Our study finding is an example of such a condition. We demonstrated that predominantly black communities were less likely to be MUAs, although they were eligible for MUAs and FQHCs. Small and McDermott found that black communities were less likely to benefit from institutional mitigation, because racial segregation and discrimination affect the level of external resources that are available through formal organizations.^37^ Our finding supports this conclusion regarding the relationship between racial segregation and resource allocation. This finding warrants intentional policies to reallocate resources and social services into highly segregated and poor communities. Broader social policy and commitment to reducing procedural barriers to safety net programs are essential to reduce the uneven distribution of MUAs. There is very little merit for the MUA designation process to be dependent on the community ability to apply for the program. MUA designation can be modified to grant the status regardless of community's ability to initiate the process.

Specific mechanisms through which race influences spatial inequality need further investigation. Wilson suggests that minority urban poverty areas became increasingly isolated. As a result, many social, economic, and/or political issues of these areas are often not shared with other neighborhoods and create difficulty in forming political coalitions.^35^ Considering the fact that obtaining MUA designation requires a certain level of civic engagement and social capital, political isolation may be one of the mechanisms that explains the decreasing likelihood of having MUAs in extremely poor minority areas. As Sampson suggests, the intersection of practices, social meanings, and spatial context may shape a differential neighborhood environment,^38^ which produces unequal outcomes for people living within such areas, consequently resulting in spatial stratification. Health disparities may constitute the most concrete disadvantages associated with the spatial and racial divide in urban areas.^36^

FQHCs are often small-scale, locally managed clinics. Consequently, FQHCs are frequently resource strained and vulnerable to financial hardship. One of the reasons that some clinics closed, whereas new clinics were opened within close proximity may be owing to a change of leadership and management. Our finding suggests the need for further investigation of how and why some FQHCs survive whereas others do not. Literature does exist on the organizational life cycle,^39–41^ but to the best of our knowledge, the life cycle of safety net organizations including FQHCs has not been examined. Research on factors contributing to the survival and longevity of FQHCs could provide valuable data to building sustainable safety net organizations.

Studies looking at neighborhood effects have focused on informal social ties, the sense of solidarity, and interpersonal trust. For example, Sampson's collective efficacy argument is that when people know their neighbors, they are more likely to engage in neighborhood affairs. Such individual-level understanding of social capital, however, limits potential solutions to improve access to primary care. As we have shown in this study, obtaining MUA designation is heavily dependent on the existence of community organizations that can mobilize a wide range of coalition among stakeholders. Community organizations play an important role in expanding social capital, particularly in resource-poor communities. Strategies to enhance formal social ties between individuals and organizations, and between organizations can greatly benefit underserved communities.

## Conclusion

Poor neighborhoods tend to have organizations that are supported by public funding that is intended to provide services to those who are poor. Consequently, poor people living in poor neighborhoods have better access to resources that are provided by these organizations. When poor people are relocated to nonpoor neighborhoods, they also lose access to community-based organizations that can mitigate individual level disadvantages. The current literature suggests that the concept of concentrated disadvantage needs to be examined in conjunction with other factors that may exacerbate or mitigate neighborhood effects.

Organizations bridge network ties and help create cross-class and cross-racial relationships. Residents in disadvantaged neighborhoods are linked to neighborhood organizations, and the organizations are linked to other wider networks of organizations, which in turn, increase residents' opportunities to access social capital that these individuals would have not possessed. This perspective provides a better way to understand urban inequality. The critical issue is why some neighborhoods can draw from these government and nongovernmental service organizations and some neighborhoods are not. Political power to advocate for one's own neighborhood is necessary to bring in needed services.

An organizational embeddedness approach makes a substantial contribution to advancing the application of social capital theory to the understanding of inequality. However, organizational embeddedness, too, is a racialized process. The presence of formal institutions, including social service agencies and nonprofit community organizations play an important role particularly in poor neighborhoods in mitigating inequality. Seemingly nonracial formal organizational interventions may produce differential results depending on the racial/ethnic composition of neighborhoods. The differential results may contribute to neighborhood disparities in health care access and outcomes.

## Abbreviations Used

ACS: American Community Survey
FQHCs: Federally Qualified Health Centers
GIS: Geographic Information Systems
HRSA: Health Resources and Services Administration
IMU: Index of Medical Underservice
MTO: Moving to Opportunity
MUAs: Medically Underserved Areas
NGOs: Non-Governmental Organizations
Non-MUA: MUA-eligible but not designated
n.s.: not significant
